# MultiSero: An Open-Source Multiplex-ELISA Platform for Measuring Antibody Responses to Infection

**DOI:** 10.3390/pathogens12050671

**Published:** 2023-05-02

**Authors:** Janie R. Byrum, Eric Waltari, Owen Janson, Syuan-Ming Guo, Jenny Folkesson, Bryant B. Chhun, Joanna Vinden, Ivan E. Ivanov, Marcus L. Forst, Hongquan Li, Adam G. Larson, Lena Blackmon, Ziwen Liu, Wesley Wu, Vida Ahyong, Cristina M. Tato, Krista M. McCutcheon, Rebecca Hoh, J. Daniel Kelly, Jeffrey N. Martin, Michael J. Peluso, Timothy J. Henrich, Steven G. Deeks, Manu Prakash, Bryan Greenhouse, Shalin B. Mehta, John E. Pak

**Affiliations:** 1 Chan Zuckerberg Biohub—San Francisco, San Francisco, CA 94158, USA; 2 Division of HIV, Infectious Disease, and Global Medicine, University of California, San Francisco, CA 94143, USA; 3 EPPIcenter Program, University of California, San Francisco, CA 94143, USA; 4 Infectious Diseases and Immunity Graduate Program, University of California, Berkeley, CA 94720-3370, USA; 5 Department of Applied Physics, Stanford University, Stanford, CA 94305, USA; 6 Department of Electrical Engineering, Stanford University, Stanford, CA 94305, USA; 7 Department of Bioengineering, Stanford University, Stanford, CA 94305, USA; 8 Department of Epidemiology and Biostatistics, University of California, San Francisco, CA 94158, USA; 9 Division of Experimental Medicine, University of California, San Francisco, CA 94110, USA

**Keywords:** serology, multiplex, ELISA, serosurveillance, open-source, SARS-CoV-2

## Abstract

A multiplexed enzyme-linked immunosorbent assay (ELISA) that simultaneously measures antibody binding to multiple antigens can extend the impact of serosurveillance studies, particularly if the assay approaches the simplicity, robustness, and accuracy of a conventional single-antigen ELISA. Here, we report on the development of multiSero, an open-source multiplex ELISA platform for measuring antibody responses to viral infection. Our assay consists of three parts: (1) an ELISA against an array of proteins in a 96-well format; (2) automated imaging of each well of the ELISA array using an open-source plate reader; and (3) automated measurement of optical densities for each protein within the array using an open-source analysis pipeline. We validated the platform by comparing antibody binding to Severe Acute Respiratory Syndrome Coronavirus 2 (SARS-CoV-2) antigens in 217 human sera samples, showing high sensitivity (0.978), specificity (0.977), positive predictive value (0.978), and negative predictive value (0.977) for classifying seropositivity, a high correlation of multiSero determined antibody titers with commercially available SARS-CoV-2 antibody tests, and antigen-specific changes in antibody titer dynamics upon vaccination. The open-source format and accessibility of our multiSero platform can contribute to the adoption of multiplexed ELISA arrays for serosurveillance studies, for SARS-CoV-2 and other pathogens of significance.

## 1. Introduction

The coronavirus disease 2019 (COVID-19) pandemic caused by SARS-CoV-2 has catalyzed the design of serological tests for antibodies against the virus. These tests have been useful in epidemiological studies that track the geographic and demographic distribution of virus infections [1,2,3,4,5]. However, many of the top-end, commercial serological assays require proprietary instruments to read the assay, and the cost of consumables, instrumentation, and analysis software can impede seroprevalence studies in resource-limited settings. Thus, despite their value, serological studies remain skewed towards high-income and upper-middle-income countries [6]. 

Multiplexed serology, in which antibody binding to multiple antigens is detected, can provide several advantages over conventional, single-antigen serological assays. These include simultaneous interpretation of the magnitude of response to multiple pathogen antigens and vaccine components [7,8,9,10], differential diagnosis of infection or exposure [11,12], and increased coverage of immunogenic epitopes [13,14,15,16,17,18,19,20]. Improved sensitivity and specificity in classifying SARS-CoV-2 seropositivity is of critical importance, given the wide range of antigen-specific antibody responses to the evolving virus [20,21,22,23,24]. Furthermore, in terms of experimental workflow, multiplexing increases the amount of information that can be acquired per volume of sera, reducing the amount of time and sera needed per antigen; however, the presence of assay-specific cross-reactivity can be a barrier to deploying highly multiplexed serological assays [25]. Nevertheless, despite the many potential benefits of multiplexed serology, uptake is limited in low-income settings due to high costs and proprietary formats compared with single-antigen antibody tests.

Here, we report on the development of multiSero, an open-source multiplex ELISA platform for analyzing antibody responses due to infection or vaccination. Built on top of a simple, plate-based ELISA format, we show that our open-source tools for image acquisition and ELISA-array quantification can compete with commercial options that can be difficult to customize and that require specialized equipment for reading the assay and analyzing the output. Our open-source work is an important step to lowering the barriers to obtaining high-content, multiplexed serosurveillance data.

## 2. Materials and Methods

### 2.1. SARS-CoV-2 Positive Samples and Negative Controls

The SARS-CoV-2 ELISA array assay was validated using plasma samples from RT-PCR-confirmed SARS-CoV-2-infected patients from the Long-term Impact of Infection with Novel Coronavirus (COVID-19) (LIINC, NCT04362150) study. For the pre-vaccine availability cohort, 93 unique samples collected from 60 individuals (10 symptomatic and hospitalized, 48 symptomatic and not hospitalized, and 2 asymptomatic) were used. For the post-vaccine availability cohort, an additional 37 samples collected from 37 individuals (29 vaccinated, 8 not vaccinated) were used. All 29 vaccinated individuals received either Comirnaty (Pfizer-BioNTech), Spikevax (Moderna), or Janssen COVID-19 vaccine (Janssen, J&J) an average of 136 days (range = 10–237 days) prior to sera donation. A total of 87 plasma samples collected before the COVID-19 pandemic were used as negative controls. All samples were stored at 4°C and diluted 1:1 in HEPES buffer (40% glycerol, 0.04% NaN3, and 40 mM HEPES in PBS), and further diluted 100X in ELISA array blocking buffer before assays. 

### 2.2. Generation of SARS-CoV-2 96-Well Plate Arrays

#### 2.2.1. S, RBD, and N Protein Production

Plasmids encoding secreted, His-tagged SARS-CoV-2 Wuhan-Hu-1 spike (S) ectodomain or receptor binding domain (RBD) [26] were transiently transfected into suspension Expi293 cells in Optimum Growth Flasks (Thomson Scientific, Toronto, ON, Canada). A total of 3 days after transfection, cell cultures at >75% viability were centrifuged at 500× *g* for 30 min, followed by filtration of the supernatant using a 0.45 μm NalGene Rapid Flow filter unit. The supernatant was adjusted to pH 7.4 and loaded onto a 5 mL HisTrap Excel column pre-equilibrated with Buffer 1 (20 mM sodium phosphate, 500 mM NaCl, pH 7.4). Captured proteins were washed with 60 column volumes (CVs) of Buffer 1 containing 20 mM of imidazole and eluted with 10 CV of Buffer 1 containing 500 mM of imidazole. Eluted proteins were buffer exchanged into PBS using either 3 kDa MWCO (for RBD) or 100 kDa MWCO (for S) Amicon concentrators and filtered through a 0.45 μm syringe filter prior to storage at −80 °C. Protein stability after freeze–thaw treatment was confirmed by analytical SEC-MALS. 

A codon-optimized His-tagged SARS-CoV-2 Wuhan-Hu-1 Nucleocapsid (N) gene [27] in a pET-28 vector (Twist Bioscience, South San Francisco, CA, USA) was transformed into T7Express bacterial expression cells (New England Biolabs, Ipswich, MA, USA) and a single colony was used to grow 10 mL of overnight culture in LB/Kanamycin, which was then used to inoculate 1 L of LB/Kanamycin. The 1 L culture was shaken at 37 °C, 200 rpm, until the OD 600 reached 0.6. The temperature was then lowered to 25 °C and protein expression was induced by the addition of 0.5 mM of Isopropyl-β-D- thiogalactoside (IPTG). After 20 h, the bacterial cells were isolated by centrifugation and the pellet was resuspended with Buffer 2 (50 mM phosphate, pH 8.0, 1 M NaCl, 10% glycerol). The cells were lysed by sonication after a 0.5 h treatment with lysozyme (MilliporeSigma, Burlington, MA, USA), benzonase nuclease (MilliporeSigma), and 1 complete EDTA-free Protease Inhibitor Cocktail (MilliporeSigma). The clarified lysate was loaded onto a 1 mL HisTrap Fast Flow column (Cytiva, Marlborough, MA, USA) at a flow rate of 1 mL/min. The column was washed with 100 column volumes (CVs) of Buffer 2 containing 50 mM imidazole and pure protein eluted in 20 CV of Buffer 2 containing 250 mM imidazole. Fractions containing pure protein were buffer exchanged using PD-10 desalting columns (Cytiva) into storage buffer (50 mM phosphate, pH 8.0, 500 mM NaCl, 10% glycerol), aliquoted, and frozen at −80 °C.

#### 2.2.2. Printing of Protein Arrays

All protein arrays were printed using a Scienion sciFLEXARRAYER S12 instrument, following general instrument parameters described previously [7]. Proteins were aspirated from a 384-well source plate (Scienion, Berlin, Germany) that was cooled and maintained at dew point and dispensed into Fluotrac™ 600, Greiner Bio-One 96-well plates (Fisher Scientific, Waltham, MA, USA) at 60% relative humidity and ambient temperature. At each position within the array, referred to as a spot, 2 or 3 330–350 pL droplets of the sample were printed. A total of 2 concentrations of SARS-CoV-2 S (62.5 μg/mL and 125 μg/mL), RBD (250 μg/mL and 500 μg/mL), and N (25 μg/mL and 50 μg/mL) proteins were printed in triplicate. Biotinylated anti-kappa light chain antibody (anti-kappa-biotin), GFP foldon, and anti-IgG Fc spots were also printed as fiducials, negative controls, and positive controls, respectively. The printed arrays were maintained at 75% relative humidity and ambient room temperature overnight to allow for the adsorption of the proteins onto the surface of the plate, followed by vacuum sealing and storage at 4 °C, which retains the quality of the antigens on the printed plate (i.e., the ability to give an ELISA signal) for several weeks.

### 2.3. SARS-CoV-2 ELISA Array Assay

Plasma samples were assayed against the 96-well SARS-CoV-2 array using conditions as described in [7] with modified parameters. Briefly, 200 μL of blocking buffer (0.05% Tween-20, 0.5% bovine serum albumin fraction V, 2% filtered fetal bovine serum, 0.2% bovine gamma-globulin, 0.05% Proclin300^TM^, 5 mM EDTA in PBS) was added to each well of the printed and cured 96-well array plates, allowed to incubate for 1 h, and then aspirated. An amount of 100 μL of serum diluted in blocking buffer was added to each well and allowed to incubate for 1 h. Diluted serum was then aspirated, and the wells were incubated for 1 h with 100 μL of blocking buffer containing 25 ng/mL each of biotinylated goat antibodies targeting the kappa (Southern Biotech, Birmingham, AL, USA cat. 2070-08) and lambda (Southern Biotech cat. 2060-08) light chains, which allows for measurement of total immunoglobulin without preference to isotype. Wells were aspirated and incubated for one hour with 100 μL streptavidin conjugated HRP (0.2 μg/mL), aspirated and developed with 50 μL sciCOLOR T2 precipitating TMB reagent (Scienion) for 10 min, and aspirated and imaged at a single time point on a Nautilus or a sciREADER CL2 plate reader. Wells were washed between steps with PBS 0.05% Tween-20. CR3022 antibody was used as a positive control anti-SARS-CoV-2 RBD antibody and was produced using methods previously described [28]. Of the 2 concentrations of each SARS-CoV-2 antigen printed, spots corresponding to ~250 pg of RBD (~1 nL of 250 μg/mL), ~62.5 pg of S (~1 nL of 62.5 μg/mL), and ~50 pg of N (~1 nL of 50 μg/mL) showed the best dynamic range of antigen-specific OD values using a dilution series of pooled SARS-CoV-2 positive sera, and therefore these antigen concentrations were used for all analyses in this study. 

### 2.4. Open-Source Plate Reader, Nautilus

The Nautilus plate reader was implemented using the Squid open-source microscopy platform [29]. In the early 2020 version of Nautilus, which was used in this study to acquire all data except for comparison of antibody responses in vaccinated and non-vaccinated cohorts, an LED backlight with a diffuser and a 650 nm bandpass filter was used for illumination and an f = 35 mm imaging lens was used as a tube lens. Manual focus was performed for well A1 before starting automated scanning of the entire plate. In an improved 2021/2022 version of Nautilus, which was used to collect data for comparison of antibody responses in vaccinated and non-vaccinated cohorts, the illumination was replaced with an RGB LED matrix (only the red channel is turned on during imaging), and an f = 50 mm imaging lens was used to provide more magnification while ensuring that the entire array in each well was covered. In addition, new motorized stages with 130 mm travel and better performance were custom designed and implemented. Motorized focus was added, however, it was not required to image our current 8 × 6 or 8 × 8 array layouts. All Nautilus units use a Jetson Nano single board computer and are controlled with the Python-based Squid graphical user interface [29], with optimizations for scanning 96-well plates. Images were saved as 8-bit bmp files for downstream processing. A Bill of Materials for Nautilus is reported in Table A1. Detailed, up-to-date information can be found at https://squid-imaging.org (accessed on 28 April 2023).

### 2.5. Multiplex ELISA Analysis with MultiSero Software 

#### 2.5.1. Detection of Spots

Given a printed microarray with known fiducial and sample grid coordinate locations, the software pipeline of multiSero extracts those locations as well as other critical parameters such as the number of rows, columns, vertical and horizontal pitch, spot width, and pixel size from the metadata.xlsx file. Spot detection is a multi-stage process beginning with several preprocessing steps that assist detection when the sample can be dirty or noisy. First, the inner-well area is extracted from the image using a combination of multi-modal intensity thresholding such as “otsu” and “rosin” thresholds, and then we check that the area meets the minimum size and eccentricity constraints. From this cropped image, we apply a uniform 2D Laplacian of Gaussian filter to the whole image using a kernel size sigma that is a quarter the spot width in nanometers. Next, this filtered image is fed into the openCV module SimpleBlobDetector [30]. The module generates a range of binarized images from thresholds based on the minimum and maximum intensities, and groups blob center coordinates across binary images. We filter out relevant blobs using the SimpleBlobDetector parameters including minimum circularity, convexity, distance between spots, and repeatability. The resulting coordinates of proposed spots are further filtered based on the distance from the border of the image.

The final step is to align the detected spots to the known printed array configuration of fiducials as defined in the metadata. Proposed spots may contain all spots in the array or may have missed some due to artifacts or lack of signal and may contain false positives from noise. To find a robust fit between fiducial locations and proposed spots we use particle filtering [31], a sequential importance sampling method often used in tracking that can also be found in medical image segmentation applications [32]. A particle is defined as a grid pose estimate, and we allow for translation, rotation, and scaling transformations when sampling particles. An initial grid estimate is based on the known physical size of the grid and is placed at the center of the image with 0 degrees of rotation and a scale of 1. A random set of N = 4000 particles is generated and for each particle the sum of distances from the fiducials to their nearest proposed spots is calculated. The weights are computed as the inverse of the summed distances and a new set of N particles is generated using importance sampling of these weights, with a small number of distortions added. This process, which allows for successful particles to proliferate while unsuccessful particles die out, is repeated until convergence.

#### 2.5.2. Measurement of OD

Once the fiducials of the array are identified using particle filtering, the expected positions of other spots can be determined by their positions on the grid. A simple threshold at 75% of pixel intensity level is performed within a bounding box around the expected spot position to segment the spot. The bounding box dimension is set to be twice as big as the expected spot width to account for spot printing and fiducial fitting errors. To robustly estimate the background across the ELISA array, the image is first divided into blocks of 128 × 128 pixels. The median of each block is computed, and a second-order 2D polynomial function is fit to the blocked median to get the background image. The spot and background intensities are then defined by the median pixel intensity of the sample and background images within the segmented spot. If a spot specified by the grid layout is not detected within the expected bounding box, a circular mask with the expected spot size and position is used to compute the local background at that spot. The OD value of a spot is defined by −log10 (intensity_spot_/intensity_background_). After computing the OD values across all wells of the plate, the multiSero software enables three types of typical ELISA analyses complete with a plotting function. These analyses are scatter plots of ODs relative to user-specified labels (e.g., positive ground truth and negative ground truth), receiver operating characteristic analysis, and four parameter logistic regressions of standard curves for interpolating antibody titers. Use of this module is through a configuration file and does not require knowledge of programming. The source code for multiSero software is available at https:/github.com/mehta-lab/multiSero (accessed on 28 April 2023).

## 3. Results

### 3.1. Overview of the ELISA Array and the Nautilus Plate Reader

**Figure 1:**
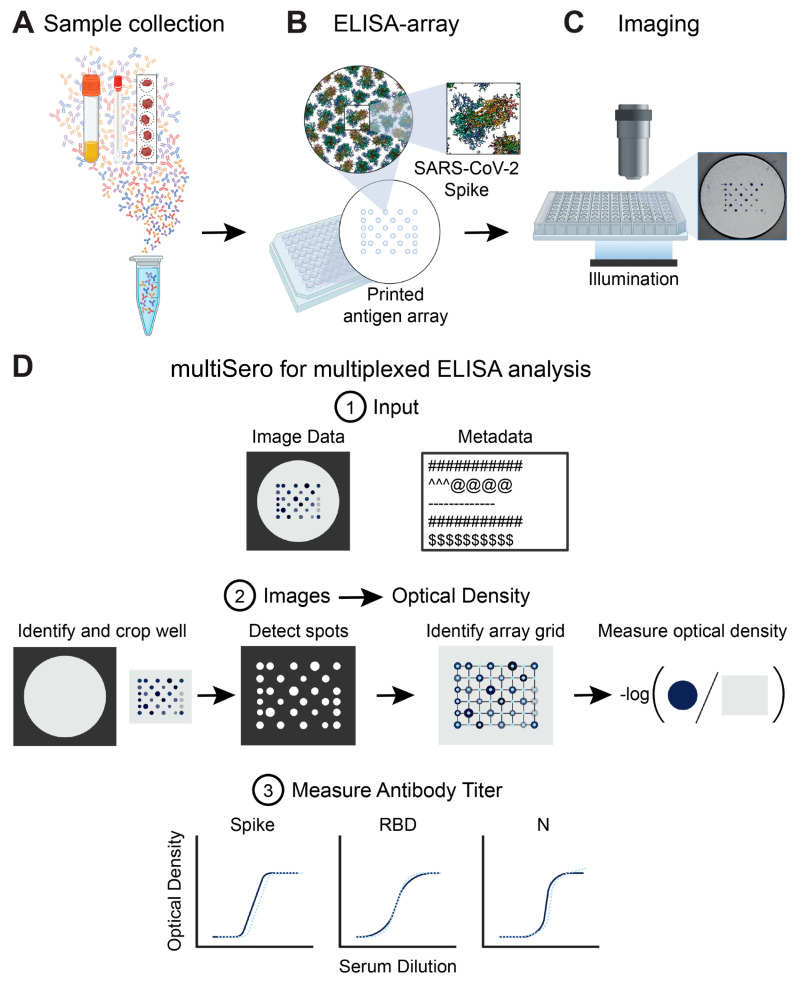
Overview of multiSero pipeline: (**A**) Samples containing antibodies derived from serum, saliva swabs, dried blood spots, or other sources are (**B**) overlaid onto an antigen array. Each spot in the array contains a concentration of a single protein or protein domain. The ELISA is performed and then the array is (**C**) imaged using the Nautilus plate reader or an alternative reader. (**D**) multiSero software is used to analyze the multi-antigen ELISA arrays. The software takes well images and a metadata file describing experimental conditions and imaging parameters as input (D1). The images are auto-cropped around the antigen array. Antigen spots are detected, and a grid is registered to the spots. Optical densities are computed from the spots that align with the registered grid. Optical density is computed as the −log of the ratio of spot intensity to the background intensity (D2). Sample antibody titers against each antigen in the array can be measured based on the ODs of control antibodies in the assay (D3).

Our multiSero assay for quantifying antibody responses to multiple antigens consists of 3 consecutive stages: (1) an ELISA against an array of proteins in a 96-well format; (2) the imaging of each well of the ELISA arrays using a plate reader; and (3) an automated software pipeline for the detection and measurement of antibody binding to each protein spot within the array (Figure 1). For the study reported here, three SARS-CoV-2 antigens (Spike (S), spike receptor-binding domain (RBD), and Nucleocapsid (N)), along with proteins to measure total IgG (anti-human IgG Fc), non-specific binding (GFP-foldon), and a fiducial marker (anti-kappa-biotin) were deposited as a grid within each well of a standard 96-well plate, using a non-contact droplet dispenser (Section 2). The picogram quantities of SARS-CoV-2 antigen deposited into each spot, which is several orders of magnitude less than that used for a conventional 96-well ELISA, were optimized to give a measurable dynamic range of COVID-19-positive sera optical densities (OD). A benefit of dispensing an array into a standard 96-well plate is that the ELISA measurement of antibody titers in sera can be performed using similar blocking, washing, and colorimetric detection protocols to that of a conventional plate-based ELISA assay (Section 2).

**Figure 2:**
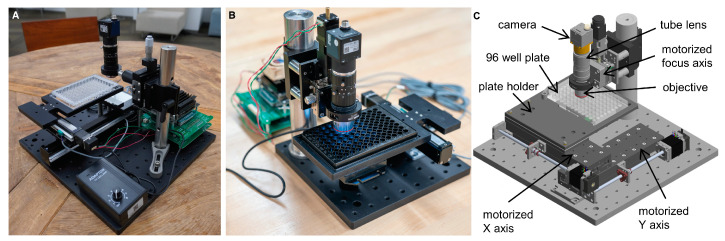
The Nautilus, an open-source 96-well ELISA-array plate reader based on the Squid platform. (**A**) An early version of Nautilus, used to acquire data shown in Figures 3–6, Figure A1, Figure A2 and Figure A3. (**B**) A smaller form-factor Nautilus unit that incorporates an optional motorized focus adjustment. (**C**) CAD of the current Nautilus unit, used to acquire data shown in Figure 7.

To image each well of the ELISA array, in a format suitable for downstream spot detection and quantification, we implemented an open-source Squid platform [29] plate reader for automated image acquisition from 96-well plates, referred to as Nautilus (Figure 2). Nautilus is low cost, has a small form factor and can run on battery power, making it suitable for scaled-up deployment in resource-limited settings. For this study, data were collected on an early version of Nautilus (Figure 2A) that requires manual focusing at well A1 prior to automated imaging of the entire plate, and a later version of Nautilus that incorporated several improvements (Section 2) (Figure 2C). 

**Figure A1:**
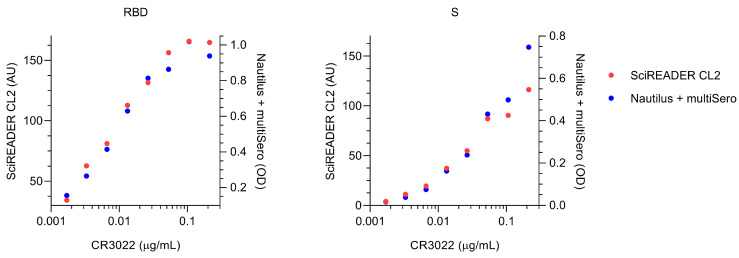
Comparison between the Nautilus reader and a commercial plate reader. The same plate and wells were imaged using Nautilus (blue) and a SciREADER CL2 (red). Median background subtracted intensity (AU, left axis) and mean OD (OD, right axis) for RBD or S against an 8-point serial dilution of monoclonal antibody CR3022 is shown.

We validated the fidelity of Nautilus-acquired images by performing an ELISA array against a serial dilution of a monoclonal anti-SARS-CoV-2 RBD antibody, CR3022 [33], followed by imaging of the same 96-well plate using both Nautilus and a commercial plate reader (Figure A1). Comparison of these data shows good agreement up to multiSero OD values of approximately 1, confirming that Nautilus is a viable, lower-cost option for ELISA-array image acquisition.

### 3.2. Analyzing ELISA Arrays

Once images of an ELISA array are acquired, spot quantification and annotation must be performed over all antigens in each well. The amount of data from a single 96-well plate, up to approximately 5700 unique serum+antigen combinations (i.e., excluding 5 spots that are used for fiducials, there can be 59 protein spots per well in an 8 × 8 grid), requires the use of an automated software pipeline to identify, annotate, and quantify spot measurements. We developed and validated open-source software to facilitate automated, high-throughput analysis (Figure 1D). A detailed description of how our software detects spots and quantifies optical density (OD) values is reported in Section 2.

**Figure 3:**
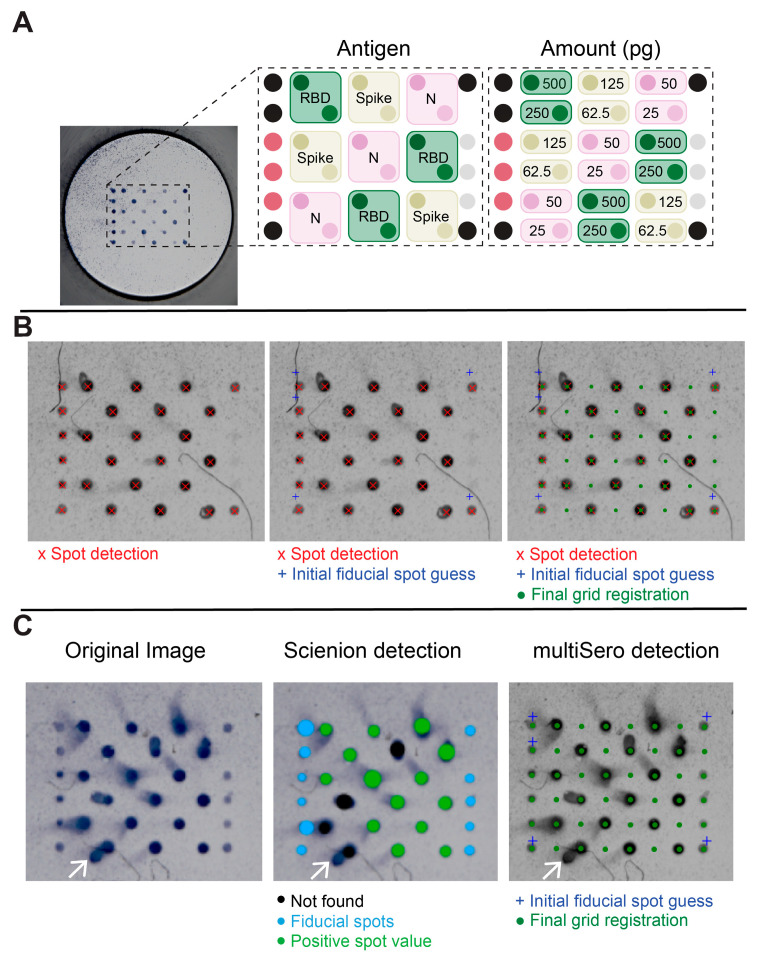
ELISA array for SARS-CoV-2 and its analysis with multiSero software. (**A**) Image of a single well of a 96-well plate and its corresponding array layout. Antigens included in the array are color coded as RBD (green), S (ochre), N (pink), anti-kappa-biotin (fiducials, black), anti-human IgG-Fc (mauve), and GFP-foldon (grey). (**B**) Grid registration for spot quantification. The center points of all spots in the cropped image (left panel) are detected first. A coordinate transformation that registers the initial guess for the fiducials (middle panel) with the detected fiducial spots is calculated. A grid containing all spot locations in the antigen layout is then transformed onto the image using the coordinate transformation (right panel). (**C**) Spot detection in the presence of comets. The original well image (left panel) shows spots that exhibit comets (white arrows). The output of Scienion spot detection and analysis (middle panel) is overlayed onto the original image, showing spots that were successfully measured (light green) or that were missed (black). The size of the spot indicates the area analyzed by Scienion. The output of multiSero spot detection and analysis (right panel) shows the final grid registration (dark green) used for spot measurement. The size of the grid registration spots are unrelated to the area analyzed.

A representative ELISA-array image of a single, uncropped well, and its corresponding protein array layout, is shown in Figure 3A. The automated, stepwise process of grid registration from a cropped image is shown in Figure 3B for an ideal well. Anti-kappa-biotin spots are important as they provide fiducial markers to estimate a coordinate transformation to overlay the detected spots onto the antigen layout; however, other positive spots (anti-IgG-Fc) were also used in those cases when the anti-kappa-biotin signal failed to provide a robust registration.

**Figure A2:**
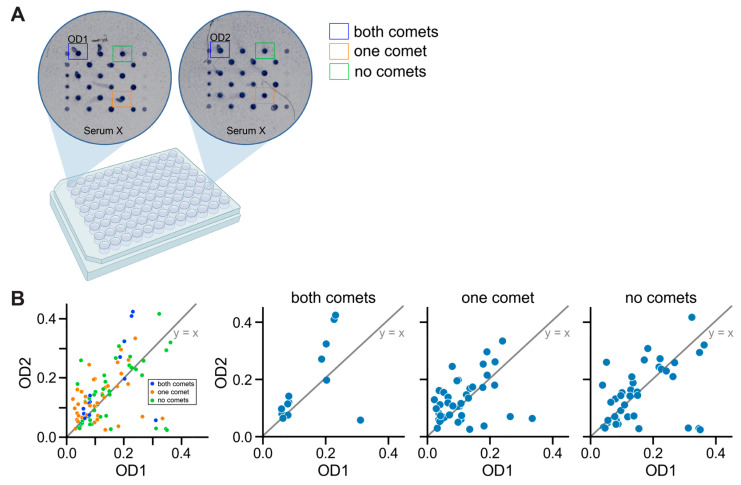
Evaluation of how comets affect measured ODs using duplicate ELISA-array wells. ODs from spots at the same locations in the array grid were compared across duplicate wells. (**A**) Schematic of example spot–spot comparison. The top-left non-fiducial spot in well A1 containing serum X was compared with the top-left non-fiducial spot in well F12 also containing serum X. (**B**) The data for one plate of duplicate sera are split according to the number of comets in the spot pairs: spot pairs in which one spot had a comet (orange); spot pairs in which both spots had comets (blue); and spot pairs in which neither spot had a comet (green). A y = x line is denoted on the plot (grey line).

Unlike a conventional ELISA, where a single antigen evenly coats an entire well, the ELISA-array format can result in the spread of a particular antigen-specific signal beyond the original spot location (Figure 3C). In the worst case these artifacts, known as comets, can overlap with adjacent spots. Careful design of the array layout, to space out antigen replicates within a well, and assaying the same serum sample over multiple wells can mitigate the effects of these artifacts. Nevertheless, the quantification of spots that have comets does pose a particular challenge for any analysis pipeline. We show that automated grid registration using multiSero software can still succeed with images that show significant amounts of comets (Figure 3C). In addition, a comparison of ODs, measured with and without comets, shows that the presence of comets is not a major source of variation in the OD measurements (Figure A2). Using the same image, the commercial (SciREADER CL2) reader’s software failed to automatically detect four spots due to comets (Figure 3C); in those cases, users can manually place a registration grid over the missed spots; however, this can be a laborious task in cases where the number of comets in a plate is high. In conclusion, the multisero software package is an automated and user-friendly software pipeline that shows robust grid registration and spot quantification, and is able to handle ELISA-array images that contain experimental artifacts (comets), all without requiring knowledge of any programming language.

### 3.3. Antigen-Specific Antibody Responses to SARS-CoV-2 Infection

**Figure A3:**
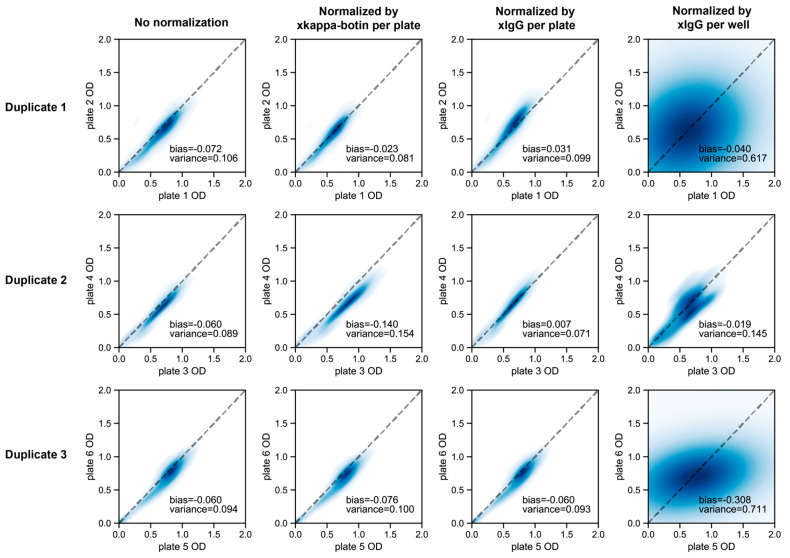
Comparison of normalization methods for correcting biases and variances in ODs across plates. Two-dimensional distribution of duplicate OD values of antibody responses of RT-PCR positive sera to SARS-CoV-2 antigens. Spots with the same array location on duplicate plates are plotted against each other and then smoothed by the kernel density function to show the density of the data points (indicated by the brightness of the blue color; high brightness indicates low density). The spot OD values were normalized by dividing the mean of the reference spot ODs (anti-IgG Fc or fiducial spot) over each plate or well as indicated at the top. Duplicates with identical spot OD values will follow the function y = x (dashed line). Performance of different normalization schemes are quantified by the bias and variance of the normalized OD values across plates, which are defined by the mean of y − x and |y − x|, respectively. A total of 3 duplicates (6 plates) are shown. Normalization in this study provided only a small improvement; nevertheless, in cases where experiments are performed with different reagent lots or over a long period of time, xkappa-biotin (per plate) and xIgG (per plate) normalization methods should be evaluated.

To measure antigen-specific antibody responses to SARS-CoV-2, we used our ELISA-array multiSero pipeline to characterize sera from two patient cohorts: a group of individuals from the Long-term Impact of Infection with Novel Coronavirus (LIINC) cohort, all of whom tested positive for SARS-CoV-2 by qRT-PCR (termed “COVID-19 positive” or “Positive” in our report), and sera acquired from a blood center that were banked before SARS-CoV-2 was circulating in the population (termed “COVID-19 negative” or “Negative” in our report). We started by restricting our analysis to samples collected before vaccine availability, from participants that reported symptoms of COVID-19 (80% not hospitalized, 17% hospitalized) or were asymptomatic (3%), to decouple antibody responses due to infection versus vaccination. Pooled SARS-CoV-2-positive, but not negative, sera showed ELISA seropositivity for SARS-CoV-2 S, RBD, and N. Individual, unpooled sera were assayed at a high concentration (1/200 dilution) to identify borderline seropositivity to SARS-CoV-2 Wuhan-Hu-1 antigens. Inter-plate normalization of replicate OD measurements was not required for analysis (Figure A3).

**Figure 4:**
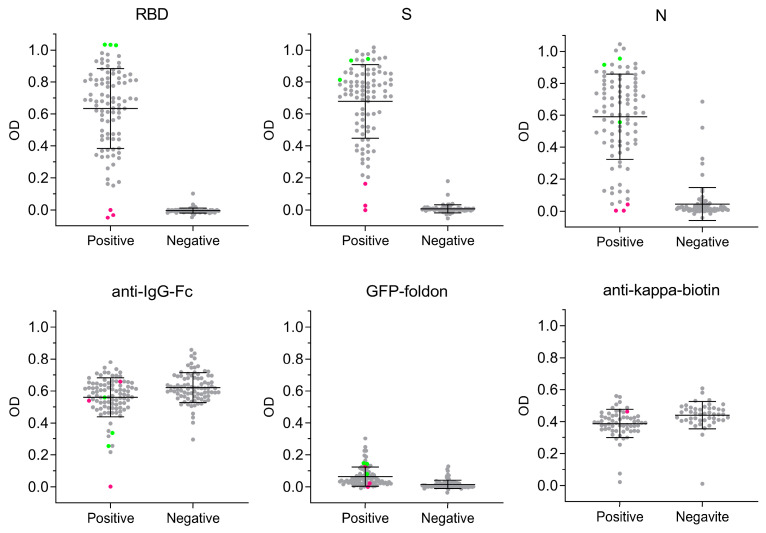
Antigen-specific antibody responses in COVID-19-positive and negative cohorts. multiSero OD values from sera obtained shortly after COVID-19 positivity, when vaccines were not available (grey dots), are shown for RBD (250 picograms (pg)), S (62.5 pg), N (50 pg), anti-IgG-Fc, GFP-Foldon, and anti-kappa-biotin. COVID-19-positive (“Positive”, 93 samples) and negative (“Negative”, 87 samples) mean OD values are plotted, along with the cohort-wide mean OD and standard deviation (black lines). The sera that showed the highest (green dots) and lowest (red dots) binding to RBD, in the COVID-19-positive cohort, are also shown for all other antigens. For anti-kappa-biotin (fiducial, secondary antibody), the single spot highlighted (red dot) corresponds to the sample that gave low antibody binding across all other antigens. All sera were assayed at a 1/200 dilution.

**Figure A4:**
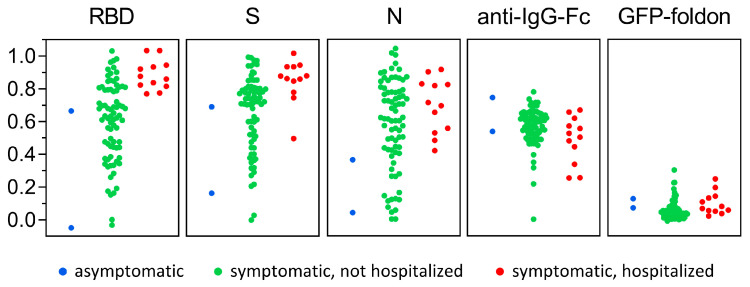
Antigen-specific antibody responses classified by COVID-19 disease severity. multiSero OD values from sera obtained shortly after COVID-19 positivity, when vaccines were not available, are shown for asymptomatic (blue), symptomatic and not hospitalized (green), and symptomatic and hospitalized (red) individuals.

The analysis of Nautilus-imaged plates showed that the COVID-19-positive cohort, relative to the COVID-19-negative cohort, had higher ODs for anti-SARS-CoV-2 antigen antibodies, while total IgG and non-specific binding (GFP-foldon) were similar between the two groups (Figure 4). Mean OD values were: (RBD, COVID-19+) = 0.63 +/− 0.25; (RBD, COVID-19-) = 0.00 +/− 0.02); (S, COVID-19+) = 0.68 +/− 0.23; (S, COVID-19-) = 0.01 +/− 0.03; (N, COVID-19+) = 0.59 +/− 0.27; (N, COVID-19-) = 0.04 +/− 0.10); (anti-IgG-Fc, COVID-19+) = 0.56 +/− 0.12; (anti-IgG-Fc, COVID-19-) = 0.62 +/− 0.09; (GFP-foldon, COVID-19+) = 0.06 +/− 0.06; and (GFP-foldon, COVID-19-) = 0.02 +/− 0.03. Antibody titers to SARS-CoV-2 antigens are associated with disease severity (Figure A4), consistent with that reported elsewhere [34].

In the COVID-19-positive cohort, 2 of 93 samples failed to show seropositivity, indicated by having an anti-RBD, anti-S, and anti-N OD value within 3 standard deviations of the mean OD for the COVID-19-negative cohort (for that specific antigen) (Figure 4). In 1 of these samples that did not seroconvert, total IgG levels were very low, 5 standard deviations below the mean OD for anti-IgG-Fc in the COVID-19-positive cohort; this individual was later vaccinated and showed robust induction of anti-RBD and anti-S antibodies (discussed below). In addition, the strong reactivity of this sample to anti-kappa-biotin (Figure 4) shows that the lack of signal across all proteins was not due to an experimental artifact of insufficient colorimetric detection substrate. For the samples that showed high antibody titers to RBD (green dots, Figure 4), there was no strong binding to GFP-foldon, showing that the strong binding to RBD is not due to non-specific aggregation. For the COVID-19-negative cohort, 8 of 87 samples showed seropositivity to N while zero samples showed seropositivity to RBD and S (at antigen-specific OD cutoffs that give 95% sensitivity), suggesting that these samples could have antibodies against endemic coronavirus N antigen that were cross-reactive with the SARS-CoV-2 N protein as measured in this assay [35].

In conclusion, the pre-vaccine availability multiSero analysis shows the value of including multiple antigens and control proteins, as it can strengthen the interpretation of edge-case samples that show borderline and/or unexpected seronegativity (false negatives) or seropositivity (false positives).

### 3.4. Sensitivity and Specificity of SARS-CoV-2 MultiSero ELISA

**Figure 5:**
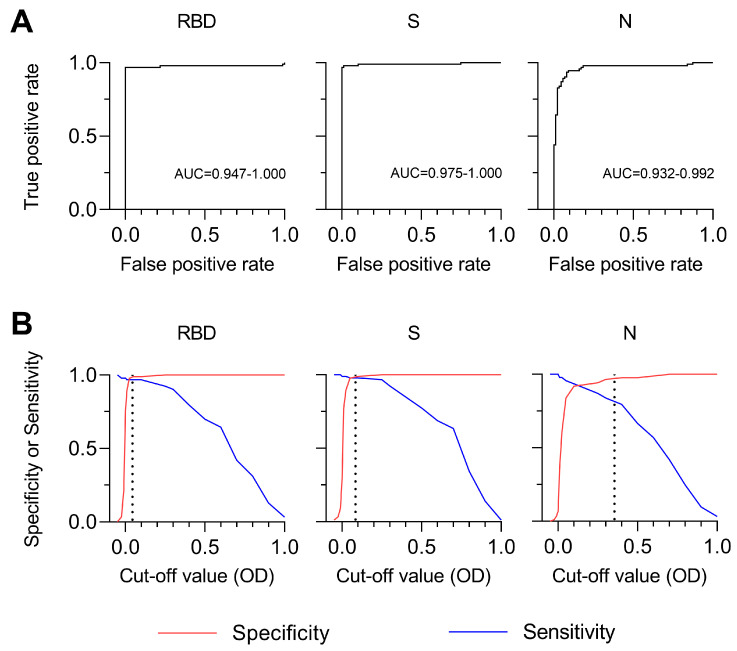
Specificity and sensitivity of antigen-specific antibody responses. (**A**) ROC curves (black line) for classifiers that use RBD, S, or N antibody responses. Confidence intervals of 95% of the area under the ROC curve (AUC) are reported below each curve. (**B**) Specificity (red line) and sensitivity (blue line) as a function of multiSero OD values. The dotted lines represent a cut-off value equal to 3 standard deviations above the COVID-19-negative mean for that antigen. Data for Figure 5 are identical to those used for Figure 4 (93 COVID-19-positive samples and 87 COVID-19-negative samples).

To determine the best antigen or antigen set for discriminating COVID-19-positive and -negative sera, we first classified sera by setting a threshold for the multiSero OD value for individual antigens (Figure 5). The classification of 180 sera (93 positive by RT-PCR and 87 negative collected before the pandemic) was used as the ground truth, and all sera were assayed at least in duplicate, spread over 6 different plates. Receiver operating characteristic (ROC) curves (Figure 5A) show that the multiSero assay is robust at classifying positivity using OD values for RBD (area under the curve, AUC, at 95% confidence = 0.947–1.000) and S (AUC 95% confidence) = 0.975–1.000), with weaker classification using OD values for N (AUC 95% confidence) = 0.932–0.992). At a cut-off value corresponding to 3 standard deviations above the COVID-19-negative sera mean OD value for that antigen (dotted line, Figure 5B), the sensitivity and specificity when using RBD, S, and N was (0.968, 0.989), (0.978, 0.977), and (0.817, 0.977), respectively, and the positive predictive value and negative predictive value was (0.989, 0.966), (0.978, 0.977), and (0.974, 0.833), respectively. Therefore, single antigen RBD or S antibody responses, as measured by multiSero, provide a robust classification of COVID-19 seropositivity, at high-sensitivity, specificity, positive predictive value, and negative predictive value. Classification performance was independent of the plate reader used, as we obtained equivalent results when using the commercial plate reader.

We evaluated methods to incorporate information from more than one antigen to classify seroconversion, including consensus-based rules as well as a gradient-boosting tree classifier [20,36,37]. While it has been shown that using a classifier combining multiple antigen-specific antibody responses can increase the accuracy of classification when individual antigens have less sensitive or specific responses [38], in our case, we did not see an improvement in sensitivity or specificity when combining multiple antigens. This is because individual RBD and S antigens already provide high accuracy for classification (Figure 5B). Using the multiSero acquired data reported here, the sensitivity of classification cannot be improved beyond the current limit of 0.978 (2 seronegative samples in 93 total positive samples) given that these samples show low OD across all antigens (Figure 4), within 3 standard deviations of the COVID-19-negative sera mean OD value for that antigen.

### 3.5. Comparison of MultiSero to Commercial SARS-CoV-2 Antibody Assays

**Figure 6:**
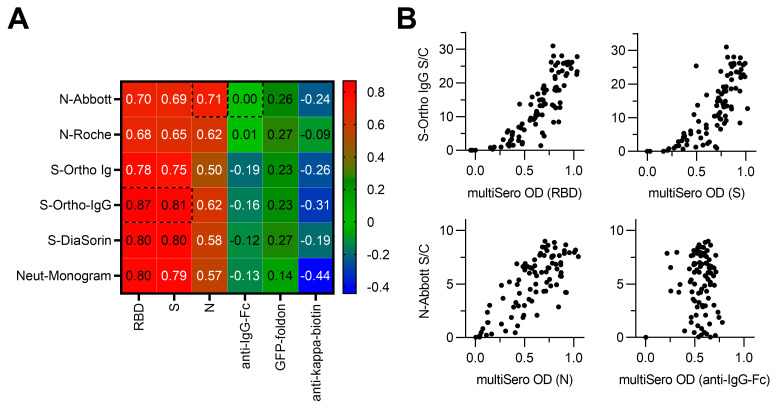
Comparison of multiSero to commercial SARS-CoV-2 antibody assays. (**A**) Heatmap of Spearman’s rank correlation between multiSero ODs and commercial SARS-CoV-2 antibody tests. Values inside the cells correspond to the Spearman’s rank correlation coefficient. Commercial assays (y-axis) are abbreviated as: Abbott ARCHITECT SARS-CoV-2 IgG (N-Abbott), Roche Elecsys anti-SARS-CoV-2 total (N-Roche), Ortho Clinical Diagnostics VITROS anti-SARS-CoV-2 total (S-Ortho Ig), Ortho Clinical Diagnostics VITROS anti-SARS-CoV-2 IgG (S-Ortho IgG), DiaSorin LIAISON SARS-CoV-2 S1/S2 IgG (S-DiaSorin), and Monogram PhenoSense Assay (Neut-Monogram). (**B**) Scatter plots of multiSero OD values and S-Ortho IgG or N-Abbott calibrator result index (S/C) values. Data shown are from the paired multiSero and commercial assay outlined in panel (**A**).

In addition to classifying positive and negative COVID-19 sera, a SARS-CoV-2 antibody assay would ideally measure relative antibody titers for antigens of interest. To determine if our multiSero results correlated with antibody levels reported by commercial SARS-CoV-2 antibody tests, we compared our antigen-specific OD values with the reported, sample-matched measurements from six commercial SARS-CoV-2 antibody assays [34]: three that measured binding of anti-S antibodies (VITROS anti-SARS-CoV-2 total, VITROS anti-SARS-CoV-2 IgG, DiaSorin LIAISON SARS-CoV-2 S1/S2 IgG); two that measure binding of anti-N antibodies (Abbott ARCHITECT SARS-CoV-2 IgG, Roche Elecsys anti-SARS-CoV-2 total); and an assay that measured neutralizing anti-S antibodies (Monogram PhenoSense Assay). multiSero OD values for RBD and S showed a strong, positive linear correlation with each commercial anti-S assay (Figure 6A), with VITROS anti-SARS-CoV-2 IgG showing the highest correlation to multiSero RBD and S, (Spearman’s rank correlation coefficients of 0.87 and 0.81, respectively). Strong, positive linear correlation was also shown for multiSero N and Abbott ARCHITECT SARS-CoV-2 IgG (Spearman’s rank correlation coefficient of 0.71). Furthermore, the two COVID-19-positive samples that did not show seropositivity by multiSero, as determined by OD values for S (Figure 4), also had very low or zero values in the commercial assays (Figure 6A), showing that the lack of detectable seropositivity is likely biological and not due to technical issues specific to the multiSero assay format. In conclusion, we validate that our multiSero pipeline can simultaneously measure RBD, S, and N antibody titers that strongly correlate with values that can be obtained by combining commercial anti-S and anti-N SARS-CoV-2 assays.

### 3.6. Antigen-Specific Antibody Responses in Vaccinated and Not Vaccinated Cohorts

**Figure 7:**
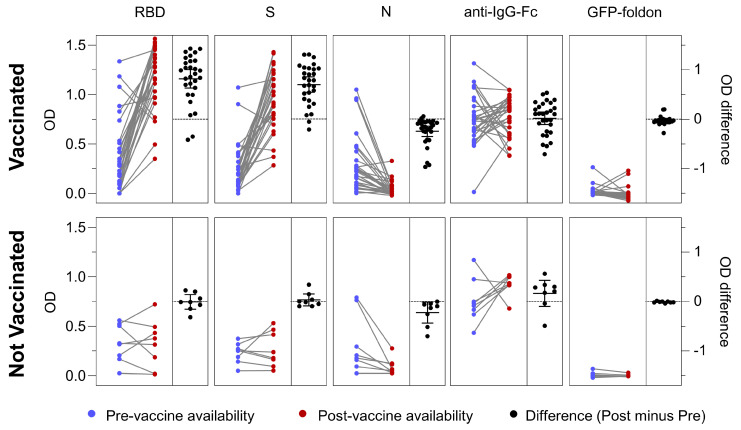
Comparison of antibody responses over time in vaccinated and not vaccinated cohorts. multiSero OD values from sera obtained shortly after COVID-19 positivity, when vaccines were not available (blue dots, “pre-vaccine availability”), and matched sera at a later time point when vaccines were available (red dots, “post-vaccine availability”) are shown for vaccinated (top panel) and not vaccinated (bottom panel) individuals. Grey lines connect matched individual sera. Black points denote matched post-availability minus pre-availability OD values, with black lines showing the mean of the difference and standard deviation. The dashed black horizonal line corresponds to zero OD difference. All sera were assayed at a 1/25,600 dilution, and the amount of each printed protein (RBD, S, N, anti-IgG-Fc, and GFP-foldon) for this analysis is identical to that of Figure 4.

The multiSero assay enables and simplifies the measurement of antibody titers for multiple antigens, which can provide an information-rich view of antigen-specific immune responses to infection and vaccination. This is of particular interest for COVID-19 given that many SARS-CoV-2 vaccines are based on S, and exclude N; therefore, anti-RBD and anti-S antibodies can arise from both infection and vaccination, whereas any anti-N antibodies would be from infection only. Here, we report antigen-specific differences in antibody responses, from baseline sera obtained shortly after COVID-19 positivity (when COVID-19 vaccines were not available) to later (12–16 month) individual-matched sera (when COVID-19 vaccines were widely available) (Figure 7). All vaccinated individuals received a vaccine that includes SARS-CoV-2 S but not N (see Section 2) and sera were collected on average 136 days post-vaccination, allowing time for induction of anti-S and anti-RBD titers. Sera were diluted to 1/25,600 (versus 1/200 for the data shown in Figure 4) to accommodate lower sample volume availability, and to improve the dynamic range between the pre- and post-vaccine time points. Images were acquired using the Nautilus reader and analyzed using multiSero software. For those individuals who received vaccination (“Vaccinated” in Figure 7, 29 samples), titers of anti-RBD and anti-S antibodies significantly increased (mean of OD difference of 0.82 and 0.70, respectively, *p* values < 0.0001), while titers of anti-N significantly decreased (mean of OD difference of −0.24, *p* value < 0.0001). In contrast, for those individuals who did not receive vaccination (“Not Vaccinated” in Figure 7, 8 samples), titers of anti-RBD and anti-S antibodies did not significantly change, while titers of anti-N significantly decreased (mean of OD difference of −0.22, *p* value < 0.04). For both cohorts, titers of total IgG and anti-GFP-foldon antibodies (negative control) were unchanged. In conclusion, multiSero analysis showed differences in antibody responses that depend on infection versus vaccination. For infection only (“not vaccinated”), anti-N antibody titers decreased and were less durable compared with anti-RBD and anti-S antibody titers over a 12–16 month time period. For vaccinated individuals (“vaccinated”), anti-RBD and anti-S antibody titers increased, while anti-N antibody titers remained less durable. Of note, the single serum that showed low total IgG and low antibody titers across all antigens tested by multiSero (Figure 4) showed an increase in anti-RBD, anti-S, and total IgG (Figure 7), revealing that this individual was capable of mounting an immune response to SARS-CoV-2 vaccination.

## 4. Discussion

The work reported here shows that open-source imaging (Nautilus) and analysis (multiSero software) can enable high-performance, commercial grade multiplexed ELISA-array analyses. For SARS-CoV-2, we show that the inclusion of multiple virus antigens and control proteins in an ELISA array can strengthen the interpretation of samples that have borderline seropositivity, with lower sample and antigen requirements compared with conventional ELISAs. For longitudinal analyses, we show that multiplexed serology can measure changes in antibody titers that are due to infections versus vaccination. Furthermore, as the COVID-19 pandemic has progressed, many additional hallmarks of disease progression and long-term sequelae, including antibody subtype [39,40], autoantibodies to type I interferons [41], cytokines [42], and co-infection with endemic pathogens [43] have been identified. Simultaneous interrogation of antibodies to these various proteins and viral epitopes in a multiplexed plate-based ELISA-array assay could further enrich the information that can be obtained from seroprevalence studies of SARS-CoV-2. In particular, the addition of antigens from SARS-CoV-2 variants, such as Omicron, is of importance for an ongoing pandemic such as COVID-19. While the study reported here measures early pandemic sera antibody titers to SARS-CoV-2 Wuhan-Hu-1 antigens, multiplexed serology using more recent sera will benefit from the inclusion of a panel of SARS-CoV-2 variant antigens, given that antibody responses can differ based on circulating variants and/or vaccine formulations [44]. 

Unique opportunities exist for the development of multiplexed serology assays tailored for public health applications, including for low- and middle-income countries (LMICs) [45]. LMICs often have a higher burden from infectious disease, as well as a more complex disease landscape, compared with other areas of the world. LMICs often face significant challenges in identifying the cause of disease, due to limited access to appropriate diagnostic tests. Serological assays have the advantage of detecting past exposure to a pathogen; thus, a serosurveillance program can help define the burden of disease from a particular pathogen within a community and help drive more informed decision making around resourcing for vaccines or therapeutics [46]. 

Immunoassays, such as ELISAs, are used to detect antibodies against specific pathogens in the serum of patients and are the workhorse of seroprevalence studies. While relatively uncomplicated, these types of assays need to be performed in the lab, are often time consuming, and typically only interrogate a single serospecificity at a time. Robust, bead-based (e.g., Luminex) multiplexed assays for SARS-CoV-2 [19,47,48,49] show similar specificity and sensitivity to multiSero, nevertheless, access to a simple plate-based multiplexed ELISA such as multiSero has the potential to make a substantial impact in infectious disease surveillance within low-resource settings [45]. Furthermore, a single serology assay that can simultaneously assess antibody responses to multiple antigen and vaccine epitopes can not only save on time and reagent costs, but can also reduce the amount of blood needed from each patient, which is a critical aspect for pediatric studies or those utilizing dried blood spots, where sample volume can be limiting.

While the multiSero ELISA-array pipeline reported here has several strengths, including robust automated analysis, accuracy, and open-source design, there can be limitations specific to the assay format and cost that should be noted. In terms of the assay format, because individual spots within the array are in close proximity within the same well, smearing of the antigen+antibody complexes (known as comets) can occur. While these comets do in fact correspond to true signals, multiSero only extracts the OD that surrounds the registered center of each spot. To ensure the most accurate measurement of ELISA-array data, it is crucial to carefully design the array to separate spots, and to optimize the blocking, wash, and protein formulation conditions to minimize artifacts. In terms of cost, the SARS-CoV-2 arrays reported here were printed onto 96-well plates using a high-precision, high-accuracy droplet dispenser. The printing technology is the most expensive aspect of the pipeline, and users without protein arrayers would need to rely on collaborators to produce and distribute the 96-well arrayed plates for serological ELISAs, imaging with Nautilus, and analysis with multiSero software. Alternative lower-cost arrays [50,51,52] could be explored given that the open-source multiSero software can be modified for use with alternative ELISA-array layouts.

Serosurveillance studies for SARS-CoV-2 and other endemic and emerging pathogens will increasingly rely on multiplexed serological tests to distinguish antibody responses to infection and vaccination, and other disease manifestations. Our open-source multiSero pipeline can be an important driver for serosurveillance studies where the measurement of these different types of antibody responses is required.

## Data Availability

The images of the SARS-CoV-2 ELISA array assayed with 180 sera, spread over 6 plates, are available publicly via Google Drive. The Python code to analyze this data is available from the multiSero GitHub repository: https://github.com/mehta-lab/multiSero (accessed on 30 March 2023).

## Appendix A

**Table A1:** Bill of Materials.

#	Description	Vendor	Part Number	Qty	Unit Price	Image Reference
	**Motorized Focus Assembly**					
1	ball bearing linear stage, stainless steel, ±6.5mm	dg-sl	LBX40-C	1	$61.14	
2	adapter for mounting SM1TC to LBX40-C	XTJ-tech		1	$25.00	
3	adapter for mounting LBX40-C to C1511	XTJ-tech		1	$25.00	
4	adapter for mounting 21H4U to LBX40-C	XTJ-tech		1	$55.00	
5	size 8 captive linear actuator, 6e-5 in/step	Haydon kerk	21H4U-2.5-A98 v1 (based on 21H4U-2.5-907)	1	$130.45	
7	Clamp for SM1 Lens Tubes	Thorlabs	SM1TC	1	$45.72	
8	SM1 Lens Tube, 0.3" threaded depth	Thorlabs	SM1L03	1	$12.52	
9	SM1 adapter for Objectives with RMS thread (Olympus, BoliOptics)	Thorlabs	SM1A3	1	$18.50	
10	M3 × 15 mm (or 14 mm) socket head screws for mounting the top plate for LBX40-C (line 2) [11.5 mm + 3.5 mm/5mm]	McMaster-Carr	91292A346 or 91292A027	4		
11	M3 × 5 mm socket head screws for mounting the LBX40-C assembly to the adapter plate for C1511	McMaster-Carr	91292A110	4		
12	8-32 × 1/2" socket head screws for mounting the assembly to C1511	McMaster-Carr	92196A194	4		
13	8-32 × 1/4" socket head screws for mounting SM1TC	McMaster-Carr	92196A190	1		
14	M2.5 × 8 mm socket head screws for adapter mounting linear actuator assembly to LBX40-C	McMaster-Carr	91292A012	2		
15	M2 × 6 mm socket head screws for mounting the linear actuator			4		
*Total: $312.19*						
	“**Microscope Body**” (may be replaced by custom machined blocks)					
1	Ø1.5" Post Mounting Clamp, 2.50" × 2.50"	Thorlabs	C1511	1	$71.57	
2	Ø1.5" Mounting Post, 1/4"-20 Taps, L = 8" (other length may be used)	Thorlabs	P8	1	$59.79	
3	Aluminum Breadboard 6" × 6" × 1/2", 1/4"-20 Taps (other size may be used)	Thorlabs	MB6	1	$51.48	
4	Ø18.0 mm Sorbothane Feet, Adhesive Mounting Surface, 4 Pieces	Thorlabs	AV3	1	$19.91	
5	1/4-20 socket head screw and washer			1		
*Total: $202.75*						
	**Imaging Lens and Camera Assembly**					
1	USB3 camera, Sony IMX 226, 1.85 um, 12 MP, 32 fps	Daheng	MER-1220-32U3M	1	$260.00	
2	f = 50 mm machine vision lens (1/1.8", f2.4, 10 MP rated)	HIKROBOT	MVL-HF5024M-10MP	1	$136.00	
3	M27 ext - SM1 int adapter for MVL-HF5024M-10MP	Thorlabs	SM1A35	1	$21.86	
*Total: $417.86*						
	**Alternative 140 mm × (up to) 140 mm stage**					
1	motorized translation stage, 80 mm travel	Kgg-robot	SSMD20-R02-080L (or Squid 130 mm travel cross roller bearing stage)	1	$150–800	
2	motorized translation stage, 140 mm travel	Kgg-robot	MVD30-RE18-200L (or Squid 130 mm travel cross roller bearing stage)	1	$150–800	
3	well plate holder (MIC-6 Al)	XTJ-tech		1	$80.00	
*(Total: $400-$1000, depending on options)*						
	**LED matrix illuminator **					
1	APA102-2020 8 × 8 RGB LED Grid	Adafruit	3444 (replaced with custom PCBA)	1	$24.95	
2	Cage Plate, for mounting the LED matrix	Thorlabs	CP37	1	$19.91	
3	Cage Plate, for condenser	Thorlabs	CP33	1	$16.89	
4	Aspheric Condenser Lens w/ Diffuser, Ø25 mm, f = 20.1 mm, NA = 0.60, 600 Grit, ARC: 350 nm–700 nm	Thorlabs	ACL2520U-DG6-A	1	$30.84	
5	Cage Rods 1" (pack of 4), for connecting the assembly	Thorlabs	ER1-P4	1	$19.77	
*Total: $112.36*						
	**Objective**					
1	4×/0.13 (0.17 coverslip correction) Plan Fluor WD 16.3mm	Boli Optics	FM13013231	1	$88.98	
*Total: $88.98*						
	**Parts in gray are commonly used hardware**					
